# Physicochemical and Microbial Diversity Analyses of Indian Hot Springs

**DOI:** 10.3389/fmicb.2021.627200

**Published:** 2021-03-03

**Authors:** Manik Prabhu Narsing Rao, Zhou-Yan Dong, Zhen-Hao Luo, Meng-Meng Li, Bing-Bing Liu, Shu-Xian Guo, Wael N. Hozzein, Min Xiao, Wen-Jun Li

**Affiliations:** ^1^State Key Laboratory of Biocontrol, Guangdong Provincial Key Laboratory of Plant Resources and Southern Marine Science and Engineering Guangdong Laboratory (Zhuhai), School of Life Sciences, Sun Yat-sen University, Guangzhou, China; ^2^Department of Pathogenic Biology, School of Basic Medical Sciences, Binzhou Medical University, Yantai, China; ^3^Henan Key Laboratory of Industrial Microbial Resources and Fermentation Technology, College of Biological and Chemical Engineering, Nanyang Institute of Technology, Nanyang, China; ^4^Bioproducts Research Chair, Zoology Department, College of Science, King Saud University, Riyadh, Saudi Arabia; ^5^Botany and Microbiology Department, Faculty of Science, Beni-Suef University, Beni-Suef, Egypt; ^6^State Key Laboratory of Desert and Oasis Ecology, Xinjiang Institute of Ecology and Geography, Chinese Academy of Sciences, Urumqi, China

**Keywords:** Indian hot springs, culture-dependent microbial diversity analysis, culture-independent microbial diversity analysis, alpha diversity, beta diversity

## Abstract

In the present study, physicochemical and microbial diversity analyses of seven Indian hot springs were performed. The temperature at the sample sites ranged from 32 to 67°C, and pH remained neutral to slightly alkaline. pH and temperature influenced microbial diversity. Culture-independent microbial diversity analysis suggested bacteria as the dominant group (99.3%) when compared with the archaeal group (0.7%). Alpha diversity analysis showed that microbial richness decreased with the increase of temperature, and beta diversity analysis showed clustering based on location. A total of 131 strains (divided into 12 genera and four phyla) were isolated from the hot spring samples. Incubation temperatures of 37 and 45°C and T5 medium were more suitable for bacterial isolation. Some of the isolated strains shared low 16S rRNA gene sequence similarity, suggesting that they may be novel bacterial candidates. Some strains produced thermostable enzymes. Dominant microbial communities were found to be different depending on the culture-dependent and culture-independent methods. Such differences could be attributed to the fact that most microbes in the studied samples were not cultivable under laboratory conditions. Culture-dependent and culture-independent microbial diversities suggest that these springs not only harbor novel microbial candidates but also produce thermostable enzymes, and hence, appropriate methods should be developed to isolate the uncultivated microbial taxa.

## Introduction

Hot springs are the places where warm or hot groundwater comes out from the Earth (Narsing Rao et al., 2018). Hot springs were once considered a sterile area (Chaudhuri et al., 2017), but Thomas Brock’s groundbreaking work in discovering *Thermus aquaticus* from a thermal environment (Brock and Freeze, 1969; Brock, 1997) has fully altered our perception of the microbial diversity of hot springs. Researchers around the world have begun to study similar ecosystems using a culture-dependent approach to understand microbial diversity (Baker et al., 2001; Pathak and Rathod, 2014; Liu et al., 2016). For decades, microbial diversity analysis was carried out by the traditional culture-dependent method; however, this method has several disadvantages. Most of the microorganisms in this method remain hidden or difficult to grow (Kumar et al., 2004; Najar et al., 2018; Narsing Rao et al., 2018).

Next-generation sequencing allows for culture-free microbial diversity detection (Sabat et al., 2017), based on molecular phylogeny of the small-subunit ribosomal RNA gene (16S rRNA gene) (Myer et al., 2016). In the past few years, this method played an important role in understanding the microbial diversity of various ecological niches (Ghelani et al., 2015; De Mandal et al., 2017; Anguita-Maeso et al., 2020).

India harbors approximately 400 geothermal springs (Pednekar et al., 2011). People bathe in these hot springs to cure diseases (Pednekar et al., 2011; Narsing Rao et al., 2018). Culture-dependent microbial diversity analysis of Indian hot springs suggested that they harbor novel candidates and produce thermostable secondary metabolites. A novel genus, *Emticicia*, was reported from an Indian (Assam) warm water spring (Saha and Chakrabarti, 2006b). A moderately thermophilic, thiosulfate-oxidizing novel species *Thiomonas bhubaneswarensis* was reported from an Atri hot spring (Bhubaneswar, India) (Panda et al., 2009). The high arsenate-tolerant novel species *Pannonibacter indica* was reported from an Athamallik (Orissa, India) hot spring sediment (Bandyopadhyay et al., 2013). Antimicrobial ability of bacterial strains isolated from a Maharashtra (India) hot spring was also reported (Pednekar et al., 2011). A novel species, *Thermus parvatiensis*, isolated from the hot water spring of Manikaran, India, was reported to produce a thermostable enzyme (protease activity at 70°C) (Dwivedi et al., 2015).

Similarly, culture-independent microbial diversity analysis of Indian hot spring samples was also carried out suggesting that they have diverse microbial diversity and that many microbial communities are still unclassified (Ghelani et al., 2015; Chaudhuri et al., 2017). Although both culture-dependent and culture-independent microbial analyses have been carried out separately to understand the microbial diversity of Indian hot springs (Kumar et al., 2004; Ghelani et al., 2015; Chaudhuri et al., 2017), only a few studies performed both analyses together (Najar et al., 2018). In the present study, we performed both culture-dependent and culture-independent microbial analyses of seven Indian hot springs. We further analyzed the various physicochemical parameters that govern microbial diversity.

## Materials and Methods

### Description of the Sample Sites and Physicochemical Analysis

**FIGURE 1:**
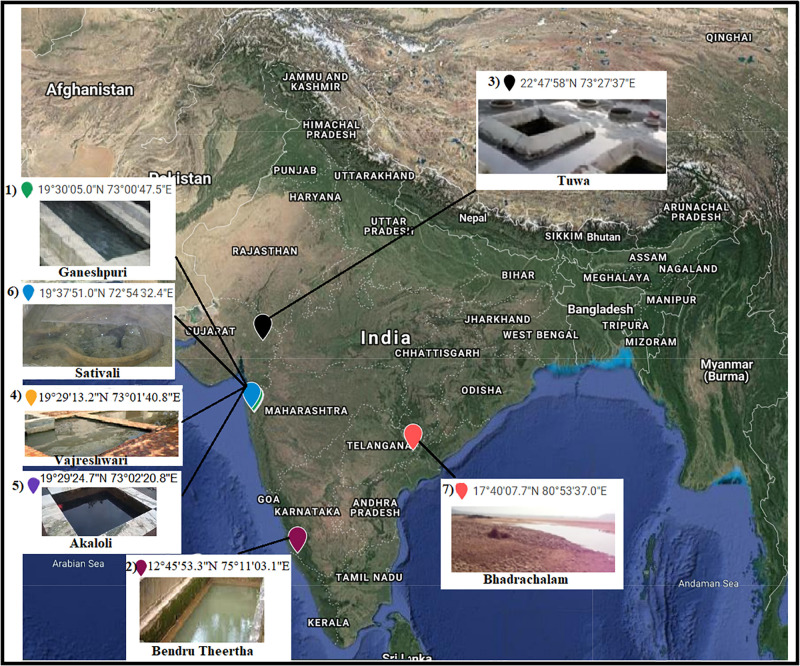
Sample sites. 1, Ganeshpuri; 2, Bendru Theertha; 3, Tuwa; 4, Vajreshwari; 5, Akaloli; 6 Sativali; 7, Bhadrachalam.

Seven hot spring samples from three Indian provinces, namely, Karnataka, Maharashtra, and Telangana, were included in the present study (Figure 1). Four hot spring samples were collected from Maharashtra Province located at Vajreshwari (VAJ) (19°29′13.2″N, 73°01′40.8″E), Akaloli (AKA) (19°29′24.7″N, 73°02′20.8″E), Ganeshpuri (GAN) (19°30′05.0″N, 73°00′47.5″E), and Sativali (SATI) (19°37′51.0″N, 72°54′32.4″E). The hot spring located in Karnataka Province is called Bendru Theertha (BT) located in the village Irde in Puttur Taluka (12°45′53.3″N, 75°11′03.1″E). The hot spring of Tuwa (TUW) (22°47′58″N, 73°27′37″E) is situated in Panchmahal District, Gujarat. The hot spring at Bhadrachalam (BHA) (17°40′07.7″N, 80°53′37.0″E), Telangana Province in Gundala is located on the bed of the Godavari River.

Water temperature and pH were measured using a portable digital thermometer and pH meter. Samples for culture-dependent analysis were collected in sterilized tubes kept in the dark, while samples for culture-independent analysis were kept in dry ice until brought to the laboratory.

### Culture-Independent Microbial Diversity and Statistical Analysis

Community genomic DNA from the sediment samples were extracted using the PowerSoil DNA isolation kit (MoBio) as per the manufacturer’s instructions. PCR was carried out by the Phusion^®^ High-Fidelity PCR Master Mix with GC Buffer (New England Biolabs) using specific primers (16S V4 region primers; 515F and 806R) (Caporaso et al., 2011) with a barcode (12 nt). The libraries were constructed using the TruSeq^®^ DNA PCR-free sample preparation kit and sequenced using the HiSeq 2500 platform (Illumina). According to the barcode sequence, data samples were separated. The paired-end reads were assembled into single reads using USEARCH 11 (Edgar, 2010). The raw reads were filtered (Bokulich et al., 2013) referring to the quality control process of QIIME 2 (Bolyen et al., 2019). Chimeras were removed using UCHIME (Edgar et al., 2011). Zero radius operational taxonomic unit (ZOTU) clustering was performed using the UPARSE pipeline (Edgar, 2013), and the taxonomic assignment was performed against the SILVA database (release 132) (Quast et al., 2013). The multisequence alignment was performed using the MUSCLE (version 3.8.31) (Edgar, 2004) software to obtain a systematic relationship of all ZOTU representative sequences. The QIIME 2 software was used to calculate the observed species (Bolyen et al., 2019), and the R software (version 2.15.3)^1^ was used to draw the species accumulation curve. The UniFrac distance was calculated using the QIIME 2 software (Bolyen et al., 2019) and the UPGMA sample clustering tree was constructed. The principal coordinate analysis (PCoA) map was drawn by the R software (see text footnote 1) using the WGCNA, stats, and ggplot2 software packages (Zhang and Horvath, 2005; Wickham, 2016). The correlation between ZOTUs was made using the sparse correlations for the compositional (SparCC) data algorithm implemented in the Python module (Friedman and Alm, 2012) with bootstraps and *p*-values of 1,000 and 0.01, respectively. The corresponding network was plotted using the R package igraph (Csardi and Nepusz, 2006).

### Culture-Dependent Microbial Diversity Analysis

According to our pre-laboratory experience of cultivating high-temperature microorganisms (Liu et al., 2016; Khan et al.,2017a,b; Habib et al., 2018), T5, R2A, ISP5, CC, TSA, and TH media were used for the isolation. Their composition details are mentioned in Supplementary Table S1.

For culture-dependent microbial diversity analysis, the samples were serially diluted. About 100 μl of the serially diluted samples were spread on six different media and incubated at temperatures of 37, 45, 55, and 70°C. The isolates obtained were picked and re-streaked on the same isolation media until pure colonies were obtained. The isolated strains were identified using 16S rRNA gene sequencing. Genomic DNA extraction and PCR amplification of the 16S rRNA gene sequence was performed as described by Li et al. (2007). The obtained 16S rRNA gene sequence was compared with available sequences of cultured species from the EzTaxon database (Yoon et al., 2017).

### Screening for Enzyme Production

The isolated organisms were screened for the presence of industrially important enzymes like amylase, protease, cellulase, and xylanase. Amylase activity was determined by inoculating bacterial strains on starch agar (Difco). The hydrolysis of starch was detected by flooding the plates with an iodine solution. The clear zone around the colony was positive for amylase activity. Protease activity was determined on skim milk agar (Sigma-Aldrich). The zone of hydrolysis around the colony was regarded as positive for protease activity.ı Cellulase activity was determined by incorporating 1% carboxymethylcellulose in tryptic soy agar (Difco). Congo red was used as an indicator for determining carboxymethylcellulose hydrolysis (Sazci et al., 1986).

### Data Availability and Accession Numbers

Raw reads were submitted to the NCBI Sequence Read Archive (SRA) under accession numbers SRR10420990–SRR10420996.

## Results

### Physicochemical Analysis of the Sample Sites

The temperature at the sample sites ranged from 32 to 67°C. The highest temperature was recorded at SATI (67°C), while the lowest was at VAJ (32°C) (Table 1). The pH at all the sample sites remained neutral to slightly alkaline (Table 1).

**TABLE 1:** Physico-chemical properties of hot springs.

Physico-chemical properties	1	2	3	4	5	6	7
pH	7.6	7.2	7.0	7.4	7.3	7.5	7.0
Temperature°C	66	38	55	32	45	67	45

**Sample site:1, Ganeshpuri; 2, Bendru theertha; 3, Tuwa; 4, Vajreshwari; 5, Akaloli; 6, Sativali; 7, Bhadrachalam.**

### Culture-Independent Microbial Diversity Analysis

**FIGURE 2:**
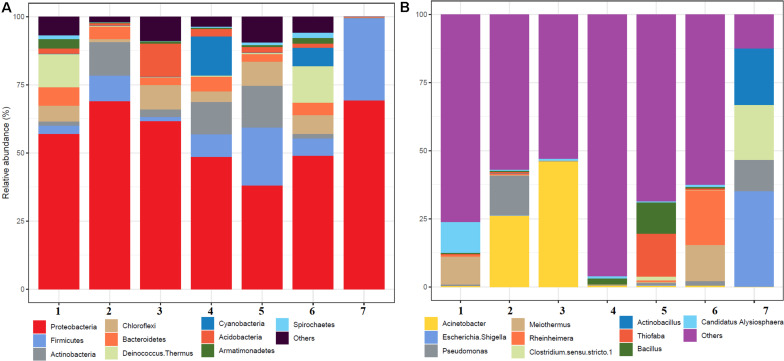
Relative abundance of the top 10 microbial communities based on the culture-independent method: **(A)** phylum level and **(B)** genus level. 1, Ganeshpuri; 2, Bendru Theertha; 3, Tuwa; 4, Vajreshwari; 5, Akaloli; 6 Sativali; 7, Bhadrachalam.

A total of 504,226 high-quality clean reads from 536,302 raw reads from seven samples were obtained and grouped into 2,965 ZOTUs. About 99.3% of ZOTUs were assigned as bacteria and 0.7% of ZOTUs were assigned as archaea. Figure 2 shows the relative abundance of the top 10 bacterial ZOTUs at different sample sites. Overall, in all the sample sites, phylum *Proteobacteria* was dominant. The other dominant phyla were *Firmicutes*, *Actinobacteria*, *Chloroflexi*, *Deinococcus*–*Thermus*, *Bacteroidetes*, *Cyanobacteria*, *Acidobacteria*, *Armatimonadetes*, and *Spirochaetes*, but their proportion in each sample varied (Figure 2A). The BHA hot spring sample had a high relative abundance of *Proteobacteria* and *Firmicutes* when compared with other samples. The GAN and SATI hot spring samples had a high relative abundance of *Deinococcus*–*Thermus*, while the VAJ and SATI samples had a high relative abundance of *Cyanobacteria* (Figure 2A). At the genus level, *Escherichia* and *Shigella* dominated the BHA sample site. *Meiothermus* dominated at the GAN and SATI sample sites. *Acinetobacter* dominated at the TUW and BT sample sites, while *Bacillus* dominated at the AKA and VAJ sample sites (Figure 2B).

**FIGURE 3:**
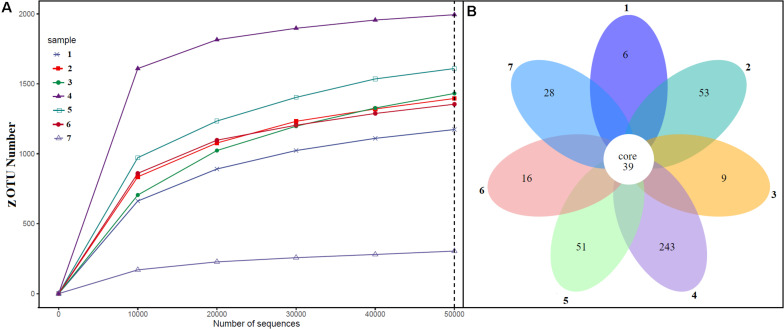
Alpha diversity. **(A)** ZOTUs per sequence number; **(B)** petal graph showing unique and common ZOTUs present in different sample sites. 1, Ganeshpuri; 2, Bendru Theertha; 3, Tuwa; 4, Vajreshwari; 5, Akaloli; 6 Sativali; 7, Bhadrachalam.

**FIGURE 4:**
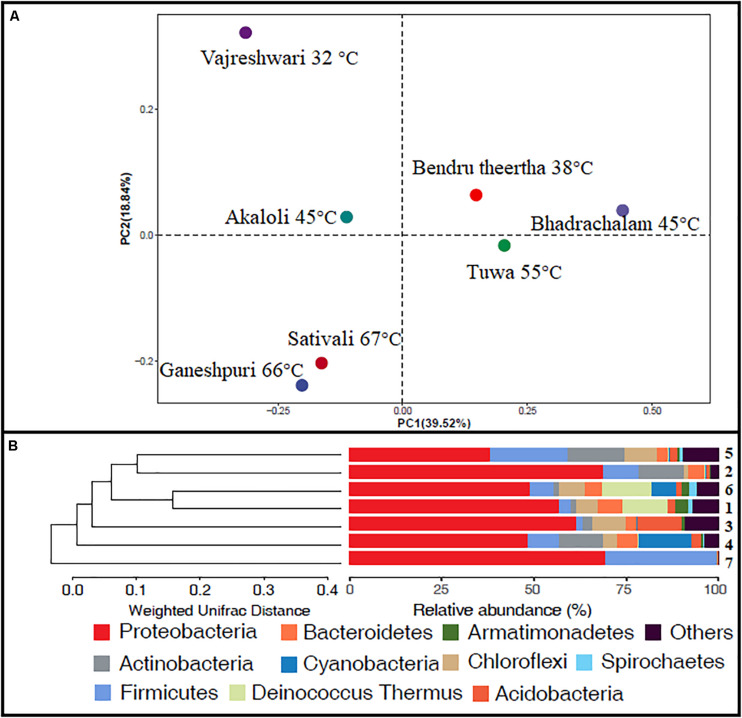
Beta diversity. **(A)** Weighted UniFrac distances calculated using amplicon reads and plotted on a PCoA plot; **(B)** hierarchical clustering based on weighted UniFrac distance (UPGMA) algorithm (1, Ganeshpuri; 2, Bendru Theertha; 3, Tuwa; 4, Vajreshwari; 5, Akaloli; 6 Sativali; 7, Bhadrachalam).

**FIGURE 5:**
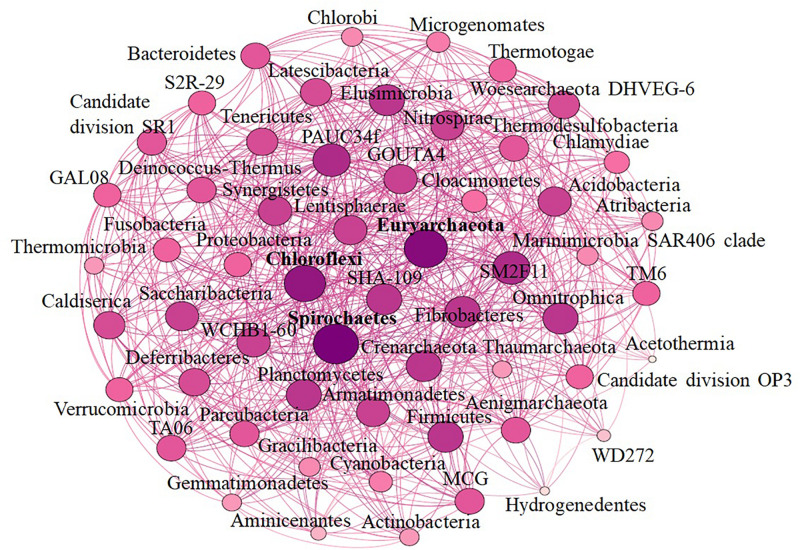
Network-based analysis.

Alpha diversity analysis showed that sample sites with low temperatures had a high taxonomic richness and the richness decreased with the increase of temperature (Figure 3A), with one exception (BHA sample site). Further, the petal diagram (Figure 3B) showed that the sample sites with low temperature had more unique ZOTUs when compared with high-temperature sample sites. Beta diversity analysis (Figures 4A,B) showed that clustering based on location (GAN, VAJ, and SATI) and their relative abundance at the phylum level were almost the same. Network analysis (Figure 5) showed that phyla *Chloroflexi*, *Spirochaetes*, and *Euryarchaeota* had a high correlation with other members.

### Culture-Dependent Microbial Diversity Analysis

A total of 131 strains (Supplementary Table S2) were isolated from the hot spring samples which were divided into 12 genera and four phyla. At the phylum level, *Firmicutes* were dominant followed by *Actinobacteria*, *Proteobacteria*, and *Bacteroidetes*. At the genus level, *Bacillus* were dominant followed by *Brevibacillus*, *Paenibacillus*, *Oceanibaculum*, *Micromonospora*, *Microbacterium*, *Terrimonas*, *Aneurinibacillus*, *Actinocorallia*, *Sphingomonas*, *Micrococcus*, and *Rhodoligotrophos.* The highest number of strains was isolated using T5 media, followed by R2A, TH, ISP5, and CC media (Supplementary Table S2). Strains SYSU-M-14, SYSU-M-111, SYSU-M1-37, and SYSU-M1-3 shared < 97% 16S rRNA gene sequence similarity with *Rhodoligotrophos appendicifer*, *Bacillus alkalitolerans*, *Paenibacillus albidus*, and *Terrimonas lutea*, respectively. It was noticed that the incubation temperature of 37 and 45°C was better for isolation. It was further noticed that no strain was isolated above 55°C. Details about the number of strains, their 16S rRNA gene sequence similarity, sample location, and media used for isolation are mentioned in Supplementary Table S2.

The isolated strains were evaluated for their ability to produce enzymes. About 63, 35, 13, and 8% of strains were positive for protease, amylase, cellulase, and xylanase, respectively (Supplementary Table S2).

### Comparison of Culture-Dependent and Culture-Independent Microbial Diversity Analysis

**FIGURE 6:**
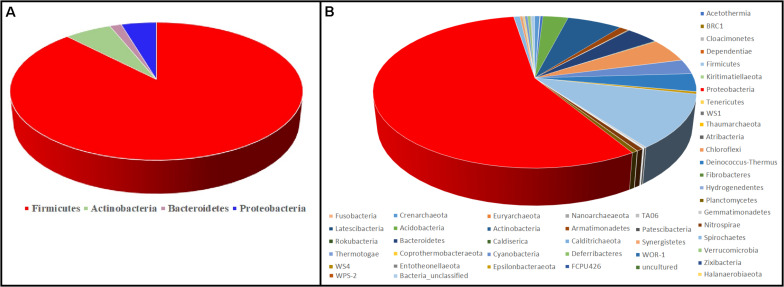
Comparison of microbial diversity at the phylum level. **(A)** Culture-dependent method; **(B)** culture-independent method.

Figure 6 represents the culture-dependent and culture-independent microbial diversity analyses of seven hot spring samples. In the culture-dependent analysis, *Firmicutes* were dominant followed by *Proteobacteria*, whereas in the culture-independent analysis, *Proteobacteria* were dominant followed by *Firmicutes.* In both culture-dependent and culture-independent analyses, the microbial richness decreased with the increase of temperature. Culture-independent analysis suggests that seven hot spring samples harbored several bacterial phyla, while only four bacterial phyla were recovered using culture-dependent analysis.

## Discussion

Hot springs were once perceived to be a sterile environment, but the pioneering work in discovering Taq DNA polymerase from *Thermus aquaticus* not only improved the economy of the industry but also brought a revolution in the field of molecular biology (Chien et al., 1976). The hot spring atmosphere was postulated to be close to the early chemical environment on Earth, and hot springs have thus become a model ecosystem for research on the origin and evolution of life (Li et al., 2015).

In the present study, physicochemical and microbial diversity analyses of seven Indian hot springs were carried out. The temperature of the seven hot springs ranged between 32 and 67°C. The highest temperature was recorded at SATI (67°C) and the lowest at VAJ (32°C). The pH of the hot spring sample ranged from neutral to alkaline which was similar to other Indian hot springs (Craig et al., 2013; Badhai et al., 2015).

*Proteobacteria* and *Crenarchaeota* (Figure 2A) were the dominant bacterial and archaeal phyla found in the present study and also in other Indian hot springs (Badhai et al., 2015; Sahoo et al., 2015; Saxena et al., 2017). Photosynthetic bacteria (*Cyanobacteria*) abundance was observed at VAJ and SATI (Figure 2A). The presence of phototrophs in the hot spring suggests the nutritive interaction among the microorganisms (Narsing Rao et al., 2018).

The GAN and SATI sample site temperatures were relatively high (66–67°C) when compared with those of the other sample sites with high abundance of *Deinococcus*–*Thermus.* Similarly, Jain et al. (2014) noticed a high abundance of *Deinococcus*–*Thermus* at the Tantloi hot spring, India, with a similar temperature. The sample sites’ temperature, ranging from 38 to 45°C (BT, AKA, and BHA), showed a high abundance of *Firmicutes* and their abundance decreased over this temperature range. At the genus level, *Bacillus* dominated at AKA and VAJ, while *Acinetobacter* dominated at the TUW and BT sample sites (Figure 2B). The abundance of the genus *Bacillus* has been reported in other Indian hot springs (Mangrola A. et al., 2015; Mangrola A. V. et al., 2015). Similarly, Saxena et al. (2017) noticed high *Acinetobacter* abundance at the Tattapani (India) hot spring. The genus *Meiothermus*, known for its thermophilic nature (Chen et al., 2002), dominated at sample sites (GAN and SATI) with a high temperature (66–67°C). The results suggest that temperature plays an important role in structuring hot spring microbial diversity.

In the BHA sample site, *Escherichia* and *Shigella* were dominant which was unusual when compared with the other sample sites in the present study. This might be due to environmental contamination as this sample site was located on the bed of a river.

Alpha diversity analysis showed that sample sites with low temperatures not only had high taxonomic richness but also had more unique ZOTUs (Figures 3A,B). In the beta diversity analysis, samples were clustered based on location (Figures 4A,B). Our results correlate with the study of Saxena et al. (2017), where the authors noticed high taxonomic richness at low temperatures and region-specific clustering.

Network analysis offers new insight into the structure of complex microbial communities, and such information is particularly valuable in environments, where the basic ecology and life history strategies of many microbial taxa remain unknown (Janssen, 2006). Further, it also helps to reveal the niche spaces shared by community members, direct symbioses between community members, potential biotic interactions, habitat affinities, or shared physiologies that could guide more focused studies or experimental settings (Proulx et al., 2005; Janssen, 2006; Barberán et al., 2012). In the present study, we noticed that phyla *Chloroflexi*, *Spirochaetes*, and *Euryarchaeota* had a high correlation with other members. Similarly, Zamkovaya et al. (2021) reported a high correlation between *Chloroflexi* and other members in the hot spring samples. Although the relative abundance of *Proteobacteria* was high (Figure 2A) when compared with *Chloroflexi*, *Spirochaetes*, and *Euryarchaeota*, however, the correlation of *Proteobacteria* with other members was less when compared with *Chloroflexi*, *Spirochaetes*, and *Euryarchaeota* (Figure 5). This suggests that the microbe’s interactions were specific irrespective of their abundance. However, further studies are needed to enlighten their interactions.

In the present study, *Firmicutes* at the phylum level and *Bacillus* at the genus level were dominant in the culture-dependent microbial diversity analysis. Similarly, Pathak and Rathod (2014) performed culture-dependent bacterial diversity analysis for the terrestrial thermal spring of Unkeshwar, India, and noticed *Firmicutes* at the phylum level and *Bacillus* at the genus level as the dominant bacterial groups.

Isolation medium and incubation temperature play an important role in determining microbial diversity through culture-dependent analysis. Pathak and Rathod (2014) during the bacterial diversity analysis of the Unkeshwar thermal spring noticed luxuriant bacterial growth on nutrient agar when compared with the other isolation medium. Sen and Maiti (2014) noticed that the optimum growth temperature for strains isolated from Odisha (India) hot springs ranged between 37 and 50°C. In the present study, the temperature range of 37 and 45°C and T5 media were better for isolation. Some of the sample sites’ temperature was above 60°C (Table 1), and culture-independent analysis showed the presence of microbial taxa (Figures 2A,B). However, we did not notice any microbial growth above 55°C using the culture-dependent method, suggesting that there are still many microbial taxa that remain uncultivated and there is still plenty of room for research.

Strains SYSU-M-14 and SYSU-M1-3 isolated from the TUW sample site and SYSU-M-111 and SYSU-M1-37 isolated from the BT sample site shared <97% 16S rRNA gene sequence similarity, suggesting that they could be novel candidates. Similarly, several novel bacterial candidates have been reported from Indian hot springs. Ruckmani et al. (2011) reported a novel genus *Calidifontibacter* from a hot spring sample of Irde (Mangalore, Karnataka State, India). Similarly, novel species *Aeromonas sharmana* and *Flavobacterium indicum* have been reported in an Indian (Assam) warm spring (Saha and Chakrabarti,2006a,c). The result suggests that Indian hot springs harbor many novel bacterial candidates. We will further characterize strains SYSU-M-14, SYSU-M1-3, SYSU-M-111, and SYSU-M1-37 to determine their taxonomic positions in our future work. The culture-dependent analysis showed that about 13 and 8% of the isolated strains were positive for cellulase and xylanase, respectively (Supplementary Table S2). Many bacterial strains isolated from Indian hot springs were reported to produce various enzymes like protease, lipase, esterase, amidase, caseinase, urease, amylase, oxidase, and gelatinase (Tripathy et al., 2016). The results suggest that Indian hot springs could be a valuable source for the enzymes.

Although the culture-dependent method is a classical approach for determining microbial diversity, this method has several disadvantages as most of the microorganisms remain hidden or are difficult to grow due to the lack of essential nutrients and optimal environmental conditions such as temperature, pH, and essential mixtures of gases (Narsing Rao et al., 2018). Culture-independent analysis suggests that seven hot spring samples harbored several bacterial phyla, while only four bacterial phyla were recovered using culture-dependent analysis (Figure 6); hence, appropriate methods should be developed to isolate these microbial taxa.

## Conclusion

Hot springs are an important source for novel strains and bioactive molecules. In the present study, physicochemical and microbial diversity analyses of seven Indian hot springs were performed. The result suggests that physicochemical parameters play an important role in shaping the microbial community of hot springs, and hence, these parameters should be considered during isolation. Culture-dependent and culture-independent microbial diversity analyses suggest that microbial richness decreases with an increase in temperature. Culture-dependent analysis suggests that these hot springs have industrially important thermostable enzymes producing bacterial strains. Culture-independent microbial diversity analysis suggests that the hot springs of the present study harbored diverse and novel microbial populations, but only a few phyla were recovered using culture-dependent analysis, and hence, appropriate methods should be developed to isolate the uncultivated microbial taxa.

## Data Availability Statement

The raw data supporting the conclusions of this article will be made available by the authors, without undue reservation.

## Author Contributions

W-JL, MX, WH, and S-XG designed the research and project outline. B-BL and M-ML performed the DNA extraction and physicochemical analysis. MN, Z-HL, and Z-YD performed the culture-dependent and culture-independent analyses. MN drafted the manuscript. All authors read and approved the final manuscript.

## Conflict of Interest

The authors declare that the research was conducted in the absence of any commercial or financial relationships that could be construed as a potential conflict of interest.

## Footnotes

1 http://www.r-project.org/
