# Supplementary material for: Physicochemical and Microbial Diversity Analyses of Indian Hot Springs
Source: Front Microbiol. 2021 Mar 3;12:627200. doi: 10.3389/fmicb.2021.627200 (PMC7982846; doi:10.3389/fmicb.2021.627200)
Supplement: Supplementary file 1 [file Table_1.docx]

**Supplementary material**

Table S1: Media composition details.

| SL. NO | Media (g L^-1^) | Composition |
| --- | --- | --- |
| 1. | T5 | Glucose 1.0, yeast extract 2.0, lotus extract 1.0, peptone 0.5, CaCO_3_ 1.0 and trace salt 1.0 ml, agar 20, the pH was adjusted to 7.0-7.2 |
| 2. | R2A | Yeast extract 0.6, peptone 0.6, casamino acids 0.6, glucose 0.6, soluble starch 0.6, sodium pyruvate 0.3, K_2_HPO_4_ 0.3, MgSO_4_ .7 H_2_O 0.05, agar 20, pH was adjusted to 7.0-7.2 |
| 3. | ISP5 | Glycerol 1.0, L-asparagine 1.0, K_2_HPO_4_ 1.0, trace salt 1.0 mL, agar 20.0 (Trace salt includes (g L^-1^): ferrous sulphate heptahydrate 0.001, manganese chloride tetrahydrate 0.001 and zinc sulphate heptahydrate 0.001. The final pH was adjusted to 7.0-7.2 |
| 4. | CC | Microcrystalline cellulose 1.0, casamino acid 1.0, KNO_3_ 0.2, Na2HPO4 0.5, MgSO_4_·7H_2_O 0.05, FeSO_4_·7H_2_O 0.01, agar 20, pH was adjusted to 7.0-7.2 |
| 5. | TSA | Soybean peptone 5.0, peptone 15.0, NaCl 5.0, agar 20, pH was adjusted to 7.0-7.2 |
| 6. | TH | Yeast extract 1.0, tryptone 1.0, nitrilotriacetic acid 100 mg, CaSO_4_. 2H_2_O 40.00 mg, MgCl_2_. 6H_2_O 200 mg, 0.01 M ferric citrate 0.50 mL, trace element solution 0.50 mL, phosphate buffer 100 mL, agar 20, pH was adjusted to 7.0-7.2 |
