# Supplementary material for: Physicochemical and Microbial Diversity Analyses of Indian Hot Springs
Source: Front Microbiol. 2021 Mar 3;12:627200. doi: 10.3389/fmicb.2021.627200 (PMC7982846; doi:10.3389/fmicb.2021.627200)
Supplement: Supplementary file 2 [file Table_2.docx]

**Supplementary material**

Table S2: List of strains isolated from different hot spring samples and their enzyme activity. VAJ, Vajreshwari; TUW, Tuwa; GAN, Ganeshpuri; SATI, Sativali; AKA, Akaloli; BHA, Bhadrachalam; BT, Bendru Theertha; N, negative; P, positive.

| SL. NO | Strains No. | Closet strain | % of similarity | Place | Media | Cellulase | | Protease | | Amylase | Xylanase | | | | Isolation temperature |
| --- | --- | --- | --- | --- | --- | --- | --- | --- | --- | --- | --- | --- | --- | --- | --- |
| 1 | SYSU-M-4 | *Bacillus altitudinis* | 100 | VAJ | R2A | N | | P | | N | N | | | | 45° C |
| 2 | SYSU-M-14 | *Rhodoligotrophos appendicifer* | 94.2 | TUW | CC | N | | N | | N | N | | | | 45° C |
| 3 | SYSU-M-18 | *Brevibacillus formosus* | 99.7 | TUW | CC | N | | N | | N | N | | | | 45° C |
| 4 | SYSU-M-20 | *Bacillus safensis* | 100 | TUW | ISP5 | N | | P | | N | N | | | | 45° C |
| 5 | SYSU-M-38 | *Bacillus altitudinis* | 100 | BHA | TSA | N | | P | | N | N | | | | 45° C |
| 6 | SYSU-M-41 | *Bacillus subterraneus* | 99.6 | AKA | TSA | P | | N | | N | N | | | | 45° C |
| 7 | SYSU-M-43 | *Bacillus safensis* | 100 | BHA | R2A | N | | P | | N | N | | | | 45° C |
| 8 | SYSU-M-58 | *Bacillus subtilis subsp. stercoris* | 99.4 | AKA | CC | N | | P | | N | N | | | | 45° C |
| 9 | SYSU-M-59 | *Bacillus subterraneus* | 99.7 | AKA | R2A | N | | N | | N | N | | | | 45° C |
| 10 | SYSU-M-60 | *Bacillus altitudinis* | 100 | VAJ | R2A | N | | P | | N | N | | | | 45° C |
| 11 | SYSU-M-61 | *Bacillus altitudinis* | 100 | AKA | R2A | P | | P | | N | N | | | | 45° C |
| 12 | SYSU-M-65 | *Bacillus pacificus* | 100 | AKA | R2A | N | | P | | N | N | | | | 45° C |
| 13 | SYSU-M-66 | *Bacillus subterraneus* | 99.5 | VAJ | R2A | N | | N | | N | N | | | | 45° C |
| 14 | SYSU-M-68 | *Bacillus subterraneus* | 99.8 | AKA | R2A | N | | P | | N | N | | | | 45° C |
| 15 | SYSU-M-70 | *Bacillus altitudinis* | 100 | AKA | ISP5 | N | | P | | N | N | | | | 45° C |
| 16 | SYSU-M-72 | *Bacillus altitudinis* | 100 | AKA | T5 | N | | N | | N | N | | | | 45° C |
| 17 | SYSU-M-74 | *Bacillus safensis* | 100 | AKA | ISP5 | N | | P | | N | P | | | | 45° C |
| 18 | SYSU-M-75 | *Bacillus safensis* | 100 | AKA | ISP5 | N | | P | | N | P | | | | 45° C |
| 19 | SYSU-M-77 | *Bacillus cucumis* | 98.3 | AKA | TH | N | | N | | P | N | | | | 45° C |
| 20 | SYSU-M-79 | *Bacillus nitratireducens* | 100 | GAN | R2A | N | | P | | N | N | | | | 45° C |
| 21 | SYSU-M-81 | *Bacillus altitudinis* | 100 | GAN | CC | N | | P | | P | N | | | | 45° C |
| 22 | SYSU-M-83 | *Bacillus altitudinis* | 100 | GAN | CC | N | | P | | N | N | | | | 45° C |
| 23 | SYSU-M-84 | *Bacillus subtilis* subsp. *inaquosorum* | 99.0 | GAN | CC | N | | P | | P | N | | | | 45° C |
| 24 | SYSU-M-85 | *Bacillus altitudinis* | 100 | GAN | TSA | N | | P | | P | N | | | | 45° C |
| 25 | SYSU-M-86 | *Bacillus infantis* | 99.4 | GAN | TSA | P | | N | | P | P | | | | 45° C |
| 26 | SYSU-M-87 | *Bacillus altitudinis* | 100 | GAN | T5 | N | | P | | N | N | | | | 45° C |
| 27 | SYSU-M-88 | *Bacillus haikouensis* | 98.8 | GAN | T5 | N | | N | | N | N | | | | 45° C |
| 28 | SYSU-M-90 | *Bacillus thioparans* | 99.5 | VAJ | TSA | N | | N | | N | N | | | | 45° C |
| 29 | SYSU-M-91 | *Bacillus altitudinis* | 100 | GAN | ISP5 | P | | P | | N | N | | | | 45° C |
| 30 | SYSU-M-98 | *Bacillus haikouensis* | 98.9 | TUW | T5 | P | | N | | N | N | | | | 45° C |
| 31 | SYSU-M-101 | *Bacillus aryabhattai* | 100 | BT | ISP5 | N | | P | | P | N | | | | 45° C |
| 32 | SYSU-M-103 | *Bacillus drentensis* | 100 | BT | ISP5 | N | | P | | N | N | | | | 45° C |
| 33 | SYSU-M-106 | *Paenibacillus cineris* | 99.1 | VAJ | ISP5 | N | | N | | N | N | | | | 45° C |
| 34 | SYSU-M-109 | *Bacillus nitratireducens* | 100 | BT | T5 | N | | N | | N | N | | | | 45° C |
| 35 | SYSU-M-110 | *Bacillus subterraneus* | 99.6 | BT | T5 | N | | N | | P | N | | | | 45° C |
| 36 | SYSU-M-111 | *Bacillus alkalitolerans* | 96.8 | BT | T5 | N | | P | | N | N | | | | 45° C |
| 37 | SYSU-M-114 | *Aneurinibacillus danicus* | 100 | BT | CC | P | | N | | P | N | | | | 45° C |
| 38 | SYSU-M-116 | *Bacillus altitudinis* | 100 | BT | CC | P | | P | | N | N | | | | 45° C |
| 39 | SYSU-M-118 | *Bacillus drentensis* | 99.4 | BT | CC | N | | N | | P | N | | | | 45° C |
| 40 | SYSU-M-119 | *Bacillus subterraneus* | 99.5 | VAJ | TSA | P | | N | | N | N | | | | 45° C |
| 41 | SYSU-M-120 | *Bacillus drentensis* | 99.4 | BT | TSA | N | | N | | N | N | | | | 45° C |
| 42 | SYSU-M-123 | *Bacillus alkalitolerans* | 97.5 | BT | R2A | N | | N | | N | N | | | | 45° C |
| 43 | SYSU-M-304 | *Bacillus flexus* | 100 | SATI | TH | N | | N | | P | N | | | | 45° C |
| 44 | SYSU-M-305 | *Bacillus paralicheniformis* | 100 | SATI | TH | N | | P | | P | N | | | | 45° C |
| 45 | SYSU-M-309 | *Bacillus flexus* | 100 | SATI | ISP5 | P | | P | | P | N | | | | 45° C |
| 46 | SYSU-M-319 | *Bacillus flexus* | 100 | SATI | T5 | N | | N | | P | N | | | | 45° C |
| 47 | SYSU-M-002 | *Micromonospora aurantiaca* | 100 | TUW | TSA | N | | P | | N | N | | | | 45° C |
| 48 | SYSU-M-003 | *Oceanibaculum indicum* | 98.8 | TUW | TSA | N | | P | | N | N | | | | 45° C |
| 49 | SYSU-M-007 | *Bacillus altitudinis* | 100 | TUW | TH | N | | P | | N | N | | | | 45° C |
| 50 | SYSU-M-019 | *Bacillus altitudinis* | 100 | TUW | T5 | N | | P | | N | N | | | | 45° C |
| 51 | SYSU-M-031 | *Bacillus subtilis* subsp. *inaquosorum* | 99.8 | BHA | CC | N | | P | | N | N | | | | 45° C |
| 52 | SYSU-M-032 | *Bacillus nitratireducens* | 100 | BHA | CC | N | | N | | N | N | | | | 45° C |
| 53 | SYSU-M-035 | *Bacillus altitudinis* | 100 | BHA | TSA | N | | P | | N | N | | | | 45° C |
| 54 | SYSU-M-037 | *Bacillus altitudinis* | 100 | BHA | TSA | N | | P | | N | N | | | | 45° C |
| 55 | SYSU-M-044 | *Bacillus nitratireducens* | 100 | BHA | R2A | N | | P | | p | N | | | | 45° C |
| 56 | SYSU-M-046 | *Bacillus altitudinis* | 100 | BHA | TSA | P | | P | | N | P | | | 45° C | |
| 57 | SYSU-M-047 | *Bacillus altitudinis* | 100 | BHA | TSA | P | | P | | N | N | | | 45° C | |
| 58 | SYSU-M-048 | *Bacillus altitudinis* | 100 | VAJ | TSA | P | | P | | P | N | | | 45° C | |
| 59 | SYSU-M-069 | *Bacillus nitratireducens* | 100 | GAN | CC | P | | N | | N | N | | | 45° C | |
| 60 | SYSU-M1-39 | *Brevibacillus formosus* | 99.0 | BT | CC | N | | N | | N | N | | | 37° C | |
| 61 | SYSU-M1-40 | *Bacillus aryabhattai* | 100 | BT | CC | N | | P | | P | N | | | 37° C | |
| 62 | SYSU-M1-41 | *Actinocorallia aurea* | 97.9 | BT | CC | N | | N | | N | N | | | 37° C | |
| 63 | SYSU-M1-133 | *Bacillus safensis* | 100 | AKA | CC | P | | P | | N | N | | | 37° C | |
| 64 | SYSU-M1-22 | *Microbacterium jejuense* | 99 | TUW | CC | N | | N | | N | N | | | 37° C | |
| 65 | SYSU-M1-23 | *Oceanibaculum indicum* | 99 | TUW | CC | N | | N | | N | N | | | 37° C | |
| 66 | SYSU-M1-123 | *Bacillus altitudinis* | 100 | TUW | CC | N | | P | | N | N | | | 37° C | |
| 67 | SYSU-M1-404 | *Bacillus altitudinis* | 100 | VAJ | ISP5 | N | | P | | N | P | | | 37° C | |
| 68 | SYSU-M1-218 | *Bacillus flexus* | 100 | SATI | ISP5 | N | | P | | P | N | | | 37° C | |
| 69 | SYSU-M1-29 | *Bacillus aryabhattai* | 100 | BT | ISP5 | N | | P | | P | N | | | 37° C | |
| 70 | SYSU-M1-30 | *Bacillus aryabhattai* | 100 | BT | ISP5 | P | | P | | P | N | | | 37° C | |
| 71 | SYSU-M1-31 | *Bacillus aryabhattai* | 100 | BT | ISP5 | N | | P | | P | N | | | 37° C | |
| 72 | SYSU-M1-32 | *Bacillus altitudinis* | 99.8 | BT | ISP5 | N | | P | | P | N | | | 37° C | |
| 73 | SYSU-M1-33 | *Bacillus drentensis* | 99.6 | BT | ISP5 | N | | N | | P | N | | | 37° C | |
| 74 | SYSU-M1-34 | *Bacillus alkalitolerans* | 96.9 | BT | ISP5 | N | | P | | N | N | | | 37° C | |
| 75 | SYSU-M1-35 | *Bacillus altitudinis* | 100 | BT | ISP5 | N | | P | | N | N | | | 37° C | |
| 76 | SYSU-M1-37 | *Paenibacillus albidus* | 94.5 | BT | ISP5 | N | | N | | N | N | | | 37° C | |
| 77 | SYSU-M1-38 | *Bacillus indicus* | 99 | BT | ISP5 | N | | N | | N | N | | | 37° C | |
| 78 | SYSU-M1-10 | *Micromonospora endophytica* | 99.2 | TUW | R2A | N | | P | | P | N | | | 37° C | |
| 79 | SYSU-M1-52 | *Bacillus aryabhattai* | 100 | BHA | R2A | N | | P | | N | N | | | 37° C | |
| 80 | SYSU-M1-107 | *Brevibacillus formosus* | 99.5 | GAN | R2A | N | | N | | N | N | | | 37° C | |
| 81 | SYSU-M1-24 | *Bacillus altitudinis* | 100 | BT | R2A | N | | P | | N | P | | | 37° C | |
| 82 | SYSU-M1-132 | *Bacillus altitudinis* | 100 | AKA | R2A | N | | P | | N | P | | | 37° C | |
| 83 | SYSU-M1-204 | *Bacillus subterraneus* | 99.7 | SATI | R2A | P | | N | | P | N | | | 37° C | |
| 84 | SYSU-M1-205 | *Bacillus paralicheniformis* | 100 | SATI | R2A | N | | P | | P | P | | | 37° C | |
| 85 | SYSU-M1-206 | *Bacillus drentensis* | 99.7 | SATI | R2A | N | | N | | P | N | | | 37° C | |
| 86 | SYSU-M1-207 | *Bacillus jeotgali* | 100 | SATI | R2A | N | | N | | P | N | | | 37° C | |
| 87 | SYSU-M1-208 | *Bacillus drentensis* | 99.2 | SATI | R2A | N | | P | | P | N | 37° C | | | |
| 88 | SYSU-M1-209 | *Bacillus safensis* | 99.7 | SATI | R2A | N | | P | | N | P | 37° C | | | |
| 89 | SYSU-M1-14 | *Bacillus nitratireducens* | 100 | TUW | T5 | N | | P | | P | N | 37° C | | | |
| 90 | SYSU-M1-53 | *Bacillus aryabhattai* | 100 | BHA | T5 | N | | P | | P | N | 37° C | | | |
| 91 | SYSU-M1-54 | *Bacillus aryabhattai* | 100 | BHA | T5 | N | | P | | P | N | 37° C | | | |
| 92 | SYSU-M1-56 | *Aneurinibacillus danicus* | 100 | BHA | T5 | N | | N | | N | N | 37° C | | | |
| 93 | SYSU-M1-59 | *Bacillus cibi* | 99.8 | BHA | T5 | N | | P | | P | N | 37° C | | | |
| 94 | SYSU-M1-119 | *Brevibacillus formosus* | 99.7 | GAN | T5 | N | | N | | N | N | 37° C | | | |
| 95 | SYSU-M1-137 | *Micrococcus aloeverae* | 100 | AKA | T5 | N | | P | | N | N | 37° C | | | |
| 96 | SYSU-M1-138 | *Sphingomonas panni* | 99.6 | AKA | T5 | N | | P | | N | N | 37° C | | | |
| 97 | SYSU-M1-212 | *Bacillus paralicheniformis* | 100 | SATI | T5 | N | | P | | P | P | 37° C | | | |
| 98 | SYSU-M1-213 | *Bacillus flexus* | 100 | SATI | T5 | N | | P | | P | N | 37° C | | | |
| 99 | SYSU-M1-215 | *Bacillus subtilis* subsp. *subtilis* | 99.6 | SATI | T5 | N | | P | | P | N | 37° C | | | |
| 100 | SYSU-M1-16 | *Terrimonas lutea* | 96.3 | TUW | T5 | N | | N | | P | N | 37° C | | | |
| 101 | SYSU-M1-17 | *Oceanibaculum indicum* | 98.8 | TUW | T5 | N | | N | | N | N | 37° C | | | |
| 102 | SYSU-M1-122 | *Brevibacillus formosus* | 99.0 | TUW | T5 | N | | N | | N | N | 37° C | | | |
| 103 | SYSU-M1-407 | *Bacillus pacificus* | 100 | VAJ | TH | N | | P | | P | N | 37° C | | | |
| 104 | SYSU-M1-55 | *Paenibacillus xylanexedens* | 99.4 | BHA | TH | N | | N | | N | N | 37° C | | | |
| 105 | SYSU-M1-102 | *Bacillus altitudinis* | 100 | GAN | TH | N | | P | | N | N | 37° C | | | |
| 106 | SYSU-M1-104 | *Bacillus pacificus* | 100 | GAN | TH | N | | P | | P | N | 37° C | | | |
| 107 | SYSU-M1-25 | *Bacillus nitratireducens* | 99.0 | BT | TH | N | | P | | N | N | 37° C | | | |
| 108 | SYSU-M1-26 | *Brevibacillus formosus* | 99.4 | BT | TH | N | | N | | N | N | 37° C | | | |
| 109 | SYSU-M1-27 | *Brevibacillus formosus* | 99.6 | BT | TH | N | | N | | N | N | 37° C | | | |
| 110 | SYSU-M1-28 | *Brevibacillus formosus* | 99.6 | BT | TH | N | | N | | N | N | 37° C | | | |
| 111 | SYSU-M1-131 | *Bacillus altitudinis* | 100 | AKA | TH | | N | P | N | | N | 37° C | | | |
| 112 | SYSU-M1-134 | *Bacillus altitudinis* | 100 | AKA | TH | | N | P | N | | N | 37° C | | | |
| 113 | SYSU-M1-135 | *Bacillus altitudinis* | 100 | AKA | TH | | P | P | N | | N | 37° C | | | |
| 114 | SYSU-M1-284 | *Bacillus subterraneu* | 100 | SATI | TH | | N | P | N | | N | 37° C | | | |
| 115 | SYSU-M1-2 | *Bacillus safensis* | 100 | TUW | TH | | N | P | N | | P | 37° C | | | |
| 116 | SYSU-M1-3 | *Terrimonas lutea* | 95.1 | TUW | TH | | N | N | N | | N | 37° C | | | |
| 117 | SYSU-M1-4 | *Micromonospora aurantiaca* | 100 | TUW | TH | | N | P | P | | N | 37° C | | | |
| 118 | SYSU-M1-5 | *Microbacterium jejuense* | 99.0 | TUW | TH | | N | P | N | | N | | 37° C | | |
| 119 | SYSU-M1-6 | *Bacillus subtilis* subsp. *inaquosorum* | 99.8 | TUW | TH | | N | P | P | | N | | 37° C | | |
| 120 | SYSU-M1-7 | *Microbacterium trichothecenolyticum* | 98.7 | TUW | TSA | | N | N | P | | N | | 37° C | | |
| 121 | SYSU-M1-108 | *Bacillus altitudinis* | 100 | GAN | TSA | | N | P | N | | N | | 37° C | | |
| 122 | SYSU-M1-109 | *Bacillus nitratireducens* | 100 | GAN | TSA | | N | P | P | | N | | 37° C | | |
| 123 | SYSU-M1-47 | *Paenibacillus lautus* | 97.8 | BT | TSA | | N | N | N | | N | | 37° C | | |
| 124 | SYSU-M1-46 | *Paenibacillus xylanilyticus* | 100 | BT | TSA | | N | N | P | | N | | 37° C | | |
| 125 | SYSU-M1-19 | *Oceanibaculum indicum* | 98.8 | TUW | TSA | | N | N | N | | N | | 37° C | | |
| 126 | SYSU-M1-200 | *Bacillus altitudinis* | 100 | VAJ | CC | | N | P | N | | N | | 37° C | | |
| 127 | SYSU-M2-8 | *Bacillus subtilis* subsp. *inaquosorum* | 99.8 | TUW | TSA | | N | P | P | | N | | 55° C | | |
| 128 | SYSU-M2-9 | *Bacillus subtilis* subsp*. inaquosorum* | 99.8 | BT | CC | | N | P | N | | N | | 55° C | | |
| 129 | SYSU-M2-17 | *Brevibacillus thermoruber* | 99.2 | BT | T5 | | N | P | P | | N | | 55° C | | |
| 130 | SYSU-M2-27 | *Brevibacillus thermoruber* | 99.5 | BHA | T5 | | N | P | P | | N | | 55° C | | |
| 131 | SYSU-M2-12 | *Bacillus thermocopriae* | 99.7 | GAN | TH | | N | P | N | | N | | 55° C | | |
